# Maximum strength and dislocation patterning in multi–principal element alloys

**DOI:** 10.1126/sciadv.abq7433

**Published:** 2022-11-09

**Authors:** Penghui Cao

**Affiliations:** Department of Mechanical and Aerospace Engineering, University of California, Irvine, Irvine, CA 92697, USA. Email: caoph@uci.edu

## Abstract

Multi–principal element alloys (MPEAs) containing three or more components in high concentrations render a tunable chemical short-range order (SRO). Leveraging large-scale atomistic simulations, we probe the limit of Hall-Petch strengthening and deformation mechanisms in a model CrCoNi alloy and unravel chemical ordering effects. The presence of SRO appreciably increases the maximum strength and lowers the propensity for faulting and structure transformation, accompanied by intensification of planar slip and strain localization. Deformation grains exhibit notably different microstructures and dislocation patterns that prominently depend on their crystallographic orientation and the number of active slip planes. Grain of single-planar slip attains the highest volume fraction of deformation-induced structure transformation, and grain with double-slip planes develops the densest dislocation network. These results advancing the fundamental understanding of deformation mechanisms and dislocation patterning in MPEAs suggest a mechanistic strategy for tuning mechanical behavior through simultaneously tailoring grain texture and local chemical order.

## Teaser

Multi–principal element alloys develop notably different dislocation patterns and deformation microstructures.

## INTRODUCTION

The strength and hardness of polycrystalline metals increase with reducing their grain size, as high stress is required for nucleation and glide of dislocations in a confined volume. The grain refinement strengthening, understood as Hall-Petch effect (*1*, *2*), can impart ultrahigh strength to materials; for example, the strength of copper exhibits a nearly two-order magnitude enhancement and achieves a gigapascal strength with decreasing the crystallite size from micrometer to nanometer scale (*3*, *4*). However, as the grain size falls into a dozen nanometers, grain boundary strengthening breaks down and softening takes over as the predominate mechanism, giving rise to the strongest size (*5*, *6*) at which the material reaches its maximum strength. The classic Hall-Petch scaling, originally proposed for pure metals, has been proved to be valid (*7*, *8*) in an emergent class of multi–principal element alloys (MPEAs). Comprising multiple principal components in high concentrations, the MPEAs, including medium- and high-entropy alloys, are presumed to be random solid solutions corresponding to maximum configurational entropy (*9*). The ideal random solid solutions in MPEAs, however, may only be possible at temperatures close to melting point (*10*). As the temperature decreases, solute-solute interaction and mixing enthalpy (enthalpic contribution) predominate the Gibbs free energy and induce local ordering of the chemistry. Appreciable short-range order (SRO) has emerged in long-time annealed MPEAs and considerably affects dislocation slip and mechanical behavior (*11*, *12*). As a salient feature pertaining to MPEAs, an intriguing question remains as to whether or how SRO affects the strongest size, maximum strength, and the underlying deformation mechanisms.

Within the numerous MPEA systems, the face-centered cubic (fcc) CrCoNi-based alloys have attracted extensive attention owing to their extraordinary mechanical properties, including high strength and ductility (*8*, *10*). The mechanistic origin of superior mechanical performance is traced to their low stacking faculty energy, which facilitates twining and phase transformation during mechanical straining. Twinning-induced plasticity and transformation-induced plasticity, known as TWIP and TRIP effects from advanced steels (*13*), provide extra interface hardening and defer the onset of necking, contributing to an enhanced strength-ductility synergy. When the SRO is engineered into the materials, both stacking fault (SF) energy and activation energy barrier of dislocation motion are increased (*14*), which likely alters the dislocation slip pathway and, hence, deformation microstructures (for instance, nanotwin and martensitic transformation). Besides the intrinsic material properties, another parameter that would control deformation microstructure of grain is its crystallographic orientation because it determines the forms of active slip system, e.g., single-planar, double-planar, and multiple-planar slip (*15*). While planar slip bands, deformation nanotwins, and deformation hexagonal close-packed (hcp) structures are widely observed in experiments (*16*–*18*), understanding the underpinning dislocation mechanism and their relationship with the grain orientation remains a challenging problem because of the inability of in-situ tracking dislocations in three-dimensional bulk materials during straining. Leveraging on large-scale deformation simulations of a model fcc CrCoNi alloy at the atomistic level, this study notably different characteristics of dislocation patterning and deformation microstructure evolution, substantially depending on grain orientation.

## RESULTS

**Fig. 1.**
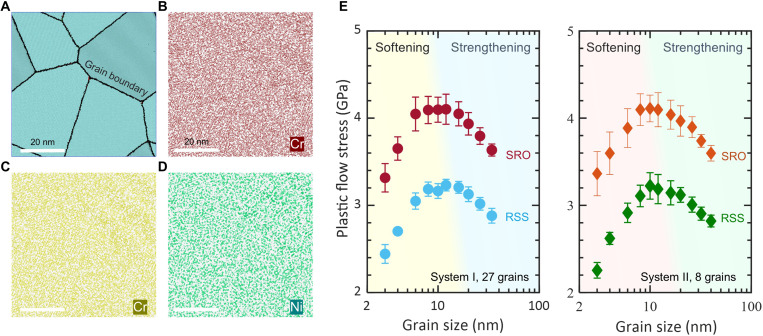
Microstructures and Hall-Petch strengthening in CrCoNi alloys. (**A** to **D**) Snapshots of the polycrystalline microstructure and the corresponding mapping of constituent elements from MC-MD annealing simulations. The detailed results of SRO analysis and element distributions in grains and grain boundaries are summarized in figs. S1 and S2. (**E**) Grain size dependence of plastic flow strength for CrCoNi alloys with RSS and SRO. The two panels correspond to two independent polycrystalline structures consisting of 27 and 8 grains, respectively.

Large-scale polycrystal models consisting of grain size from 40 to 3 nm and involving up to 97.3 million atoms are adopted. We use hybrid Monte Carlo (MC) and molecular dynamics (MD) simulation to obtain equilibrated systems with SRO (see Materials and Methods); hereafter, we refer to SRO and RSS as polycrystalline systems with short-range order and random solid solution, respectively. Figure 1 (A to D) shows the structure and chemical mapping of an annealed polycrystal with a grain size of 32 nm. Note that we perform the same number of MC swap trials in all system (grain) sizes, which leads to a slight variation in the degree of chemical order. Nevertheless, the detailed analysis on chemical distribution and local order in all models confirms the same trend of chemical SRO in grains and grain boundaries (figs. S1 and S2). Under the same annealing procedure, SRO systems with local chemical order are generated to compare with their RSS counterparts, aiming to understand the impact of introduced SRO on mechanical behavior. Subjected to uniaxial tensile deformation, the stress-strain responses of polycrystals display an initial elastic regime followed by plastic flow (fig. S3). Fig. 1E demonstrates the variation of plastic flow strength of polycrystals with their constituent grain size. The grain size dependence of strength reveals that the maximum strength of SRO systems is appreciably raised as compared to RSS. The critical grain size (strongest size) corresponding to the highest strength drifts to a smaller value in SRO. This suggests that the introduced chemical SRO strengthens the materials, stabilizes grain boundaries, and extends the Hall-Petch effect to smaller nanograins.

**Fig. 2.**
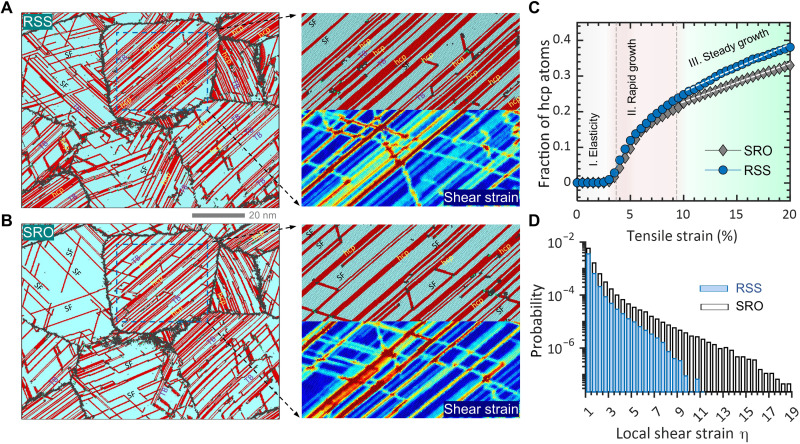
Deformation microstructure and local plastic strain. (**A** and **B**) Atomic structures and local strain for RSS and SRO samples stretched to 20% strain, respectively. Atoms are color-coded by structural type, where the red color represents the hcp-coordinated atoms and blue indicates the fcc structure. (**C**) Volume fraction of hcp-coordinated atoms as a function of tensile strain. (**D**) Probability distributions of local strain in RSS and SRO. The SRO system exhibits long tail and extreme local strain after deformation.

To understand the effects of local chemical order on deformation mechanisms, Fig. 2 (A and B) shows the typical deformation microstructures at 20% strain for RSS and SRO samples, respectively. A high fraction of hcp-coordinated atoms appears in the fcc grain and manifest in the form of SF, hcp phase, and twin boundary (TB). The morphology of these hcp-type structures varies from one grain to another, indicating grain orientation dependence of deformation substructures (to be discussed later). In contrast with RSS, the SRO sample exhibits a reduced fraction of hcp atoms. As can be seen in Fig. 2C, the volume fraction of deformation-induced hcp atoms is plotted as a function of tensile strain. The variation of hcp fraction with strain demonstrates three-stage behaviors, referred to as incubation period in elastic deformation regime, followed by rapid growth after yielding, and, last, the steady and slow growth during plastic flow stage. At 20% tensile strain, the SRO system accumulates 33 volume % of hcp structure, while RSS reaches more than 38% volume fraction. The decrease of hcp atom fraction in SRO is closely related to SRO-induced SFE increase (*14*, *19*) and glide plane softening (*20*), as the former lowers the propensity for nucleation and growth of hcp lamellae. The latter resulting from local SRO destruction promotes repetitive partial dislocation slip in the slip-softened layers such as SF and hcp lamella. Because no footprint (defects) will be left over in the grains after successive passage of leading partial and trailing partial, the instantaneous deformation structure is unable to trace the deformation history that the sample has experienced. To evaluate plastic deformation strain localization, we calculate the atomic-level plastic strain η (*21*), and its spatial distributions are shown in the right panels of Fig. 2 (A and B). In comparing the strain maps, one can see that SRO exhibits extended and strain-concentrated planar slip nanobands, implying that repetitive dislocation glide takes place in the band regions. The increased strain localization, originating from SRO destruction and glide plane softening, is quantitatively depicted in Fig. 2D, in which the statistical distributions of large local strain (η > 1) are shown. From the extreme value theory aspect (*22*), the SRO material carrying large plastic strain bursts (the long tail in the η distribution) is prone to cause incompatible deformation and initiate decohesion events (nanocrack formation).

**Fig. 3.**
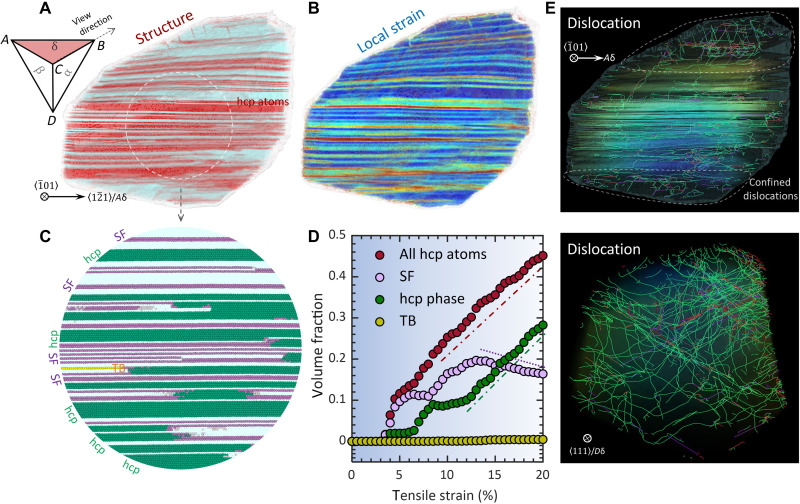
Deformation characteristics and dislocation pattern in grain of a single active slip plane. (**A** and **B**) Deformation microstructure and local strain map of grain deformed at 20% strain, respectively. Thompson tetrahedron is shown to illustrate active slip plane *ABC* and view direction *CB*. The hcp-coordinated atoms are colored in red. (**C**) Magnification of the deformation microstructure in the circled region in (A). The hcp-coordinated atoms are identified as SF, hcp phase, and TB. (**D**) Volume fraction of deformation structures as a function of strain for the grain with single planar slip. (**E**) Dislocation configurations in the deformed grain. Smoothly curved Shockley partial dislocations (green) lie in the parallel *ABC* planes, and stair-rod (purple) and Hirth partial (red) dislocations are scattered.

Governing by grain orientation and Schmid’s law, a different number of slip planes can be activated under mechanical straining and substantially influence, if not redetermine, dislocation patterns and deformation microstructures. Figure 3 presents a deformed grain with a single primary slip plane activated, where deformation microstructure, local strain map, and dislocation configurations are delineated. The smoothly curved and long Shockley partial dislocations, emitted from grain boundary, glide on parallel (111) planes (i.e., triangle *ABC* in Thompson tetrahedron) without intersection (Fig. 3E). The resulting local strain is uniformly spreading, crossing these planes (Fig. 3B). Arising from partial dislocation glide, SF is produced behind a moving leading partial in fcc structure. The incipient hcp phase nucleates when two partials glide on every other (111) plane. The deformation microstructure (Fig. 3A) exhibits piles of hcp lamella and SF [including intrinsic SF (iSF) and extrinsic SF (eSF)] as detailed in Fig. 3C. These deformation-induced hcp structures impede dislocations on other intersecting slip planes (benefiting from grain rotation during deformation), confining them in the grain corners (Fig. 3E). Fig. 3D presents the strain variation of the volume fraction of all hcp-coordinated atoms (red-colored circles) that consist of SF (purple), hcp phase (green), and TB (yellow). In the early stage of plastic deformation (5% strain), the hcp structures are predominantly SFs, and they rapidly grow with strain. From 10% strain, hcp phase growth, i.e., martensitic transformation (fcc to hcp), turns into the primary deformation mode which consumes the existing SFs (decrease of SF in Fig. 3D). At 20% strain, 45 volume % of the fcc phase is transformed to hcp structures manifested as hcp phase/lamella (28%), SFs (16%), and TB (1%) (see movie S1 for microstructure and dislocation evolutions).

**Fig. 4.**
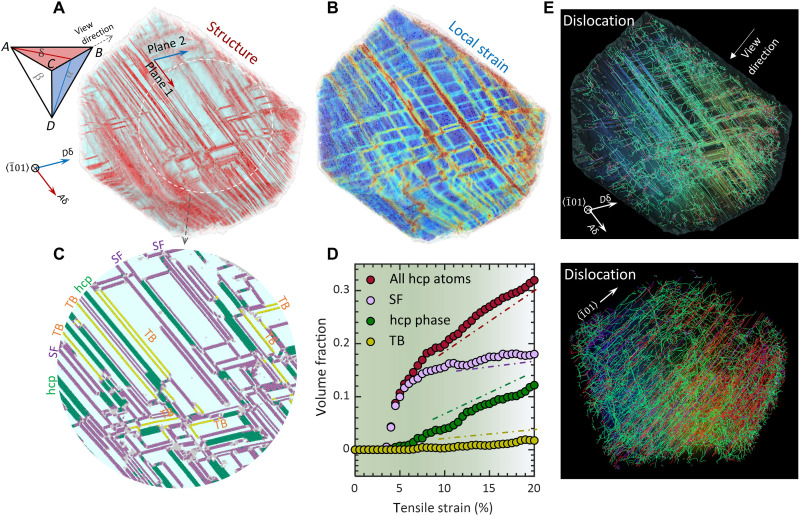
Deformation characteristics and dislocation pattern in grain of double active slip planes. (**A** and **B**) Deformation microstructure and local strain map of grain at 20% strain, respectively. Thompson tetrahedron is shown to illustrate active slip planes *ABC* and *BCD*. (**C**) Magnification of deformation microstructure in the dashed circle region in (A). The hcp-coordinated atoms are characterized as SF, hcp phase, and TB. (**D**) Volume fraction of deformation structures as a function of strain for the grain with double-planar slip. (**E**) Dense dislocation network in the deformed grain. Highly curved Shockley partial dislocations (green) lie in two intersecting slip planes, and dense stair-rod (purple) and Hirth partial (red) dislocations appear in the grain interior.

The grain having two active slip planes exhibits distinct deformation microstructure and dislocation patterns (Fig. 4). Dislocations that glide on two intersecting planes of (111) and $\left(\bar{1}1\bar{1}\right)$ (i.e., *ABC* and *BCD* in Thompson’s notation) collide into each other. Their reaction generates a high density of sessile dislocations: stair-rod partial and Hirth partial (Fig. 4E), depending on the direction of Burgers vectors (*23*). For example, two partial dislocations γ*B* and *B*δ can react to form a stair-rod dislocation γδ, $\frac{1}{6}[\bar{1}21]+\frac{1}{6}[2\bar{1}\bar{1}]=\frac{1}{6}[110]$; the reaction of partials γ*B* and δ*A* results in formation of Hirth partial, $\;\frac{1}{6}[\bar{1}21]+\frac{1}{6}[1\bar{2}1]=\frac{1}{3}[001]$ (see fig. S5 for detailed reaction process).Owing to the dislocation slip, the grain is gridded and develops an hcp structure network (Fig. 4A). Note that TBs are always associated with intersection lines (Fig. 4C), implying that twinning dislocation is mediated by collision and reaction (*24*). Compared with the grain of a single planar slip, the current grain features a faster accumulation of hcp atoms right after yielding (5% strain in Fig. 4D) because of simultaneous slip on two planar systems. However, during the plastic flow stage, the growth of the hcp structure is slower and reaches 32% volume fraction at 20% strain (structure volume and dislocation density summarized in table S1). Concerning individual hcp-type structures, SFs become saturated at 10% strain. In the meantime, both hcp phase and TB begin to progress rapidly, leading to martensitic transformation and twinning deformation in plastic flow (movie S2).

**Fig. 5.**
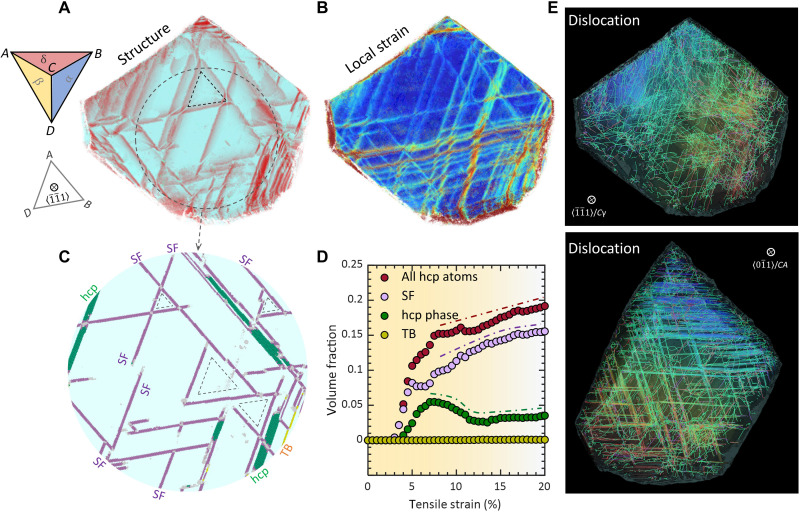
Deformation characteristics and dislocation pattern in grain of multiple active slip planes. (**A** and **B**) Deformation microstructure and local strain map of grain at 20% strain, respectively. (**C**) Magnification of deformation microstructure in the dashed circle region in (A). The hcp-coordinated atoms are mainly SFs. (**D**) Volume fraction of deformation structures as a function of strain for the grain with multiplanar slip. (**E**) Dislocation network in the deformed grain shows some local regions with concentrated dislocations. Shockley partial dislocations (green) lie in multiple slip planes, with variously arranged stair-rod (purple) and Hirth partial (red) dislocations.

Figure 5 shows the deformation microstructure and dislocation configurations in a grain of multiple slip planes activated in deformation. From the structure aspect, the deformation-induced hcp slices, lying in their own slip planes, intersect and form three-dimensional networks (Fig. 5, A and C). Examining microstructure evolution (Fig. 5D), we can see that after yielding, a large fraction of hcp structures is formed but immediately becomes saturated during plastic flow. The entire hcp structures, which are mainly SFs, occupy less than 20% of the grain volume at the end of deformation. Twinning deformation is unfavored when multiplanar slippage is activated, and martensitic transformation is appreciably suppressed, as indicated by the reduction of hcp phase in the plastic flow stage (7 to 10% strain in Fig. 5D). As a result of dislocation motion on multiple intersecting planes, a dense dislocation line network composed of glissile and sessile dislocations is developed (Fig. 5C). These sessile stair-rod dislocations and Hirth partials bridge Shockley partials on various slip planes, presumably acting as the backbone of dislocation network (movie S3).

## DISCUSSION

At a given temperature and strain rate, the strength of MPEAs is dictated by dislocation motion–related solid solution strengthening and grain size–dependent Hall-Petch effect. In the presence of SRO and, consequently, high SFE (*14*, *19*), it is reasonable to speculate that the motion of leading partial will experience a large resistance and high energy barrier. In addition, the following passage of trailing partial, when it happens, can erase the SF and restore the fcc structure but inevitably create a diffuse antiphase boundary owing to the destruction of SRO on the slip plane. The interplay between SF creation, antiphase boundary generation, and the associated energy penalty, stemming from the existence of SRO, exerts back stress on moving dislocation and retards its motion, which is the root cause of enhanced solid solution strengthening. The Hall-Petch strengthening is influenced by SRO in a way that the critical grain size (strongest size) corresponding to the maximum strength decreases to a smaller value, accompanied by deferred strength softening (Fig. 1E). This implies that grain boundary–mediated plasticity, such as boundary sliding and migration responsible for the softening (*6*), is alleviated by the introduced SRO. The mitigation of boundary softening benefits from SRO-lowered boundary enthalpy (fig. S7) that improves its sliding resistance. As evidenced in fig. S8, the grain boundary sliding and migration, quantified by non-affine displacement (*6*), are considerably alleviated by the introduced SRO. The SRO in the grain interior hinders grain boundary migration because, when a grain boundary sweeps its grain matrix, the chemical SRO in the swept volume will be destroyed as the boundary migration alters the crystallographic orientation of the swept matrix (*25*). The grain size–dependent chemical ordering in grain boundary and matrix stabilizes boundary network and mitigates softening effects, deserving careful attention and further study. Note that the imposed straining rate (10^8^ s^−1^) in the simulations, which can adequately enable true dislocation dynamics (*26*), is still several orders of magnitude higher than that of conventional laboratory experiments. When sufficiently lowering the strain rate, thermally activated diffusional processes, including vacancy-mediated dislocation climb and Coble creep, can take place and decrease the strength. Because chemical SRO considerably reduces and localizes diffusion (*27*), it is tempting to hypothesize that the strength difference between RSS and SRO systems will widen even further at reduced strain rates and/or elevated temperatures.

Essentially rooted in dislocation motion, deformation microstructure evolution and dislocation pattern formation have remained as the central focus of physical metallurgy to interpret and ultimately predict mechanical behavior. The ultralarge atomistic simulations, capturing the individual atomic motions, enable the study of dislocation motion and patterning at the fundamental level. In particular, it elucidates the atomistic processes of deformation twinning and martensitic transformation that the other mesoscale simulation methods (*28*) such as discrete dislocation dynamics are inadequate to conquer. Owing to the SRO imparted to the system and resulting high SFE and antiphase boundary generation, different deformation processes, including dislocation slip, faulting, twinning, and phase transformation, are influenced and balanced so that the system deforms along its minim energy pathways. We found that, in the existence of local chemical order, faulting and deformation transformation are suppressed and planar slip is increased (glide plane softening and repetitive slip), which gives rise to a decrease of hcp structure density and exacerbation of strain localization in SRO alloys. Another factor responsible for the strain large localization is associated with dislocation multiplication. The SRO system forms bundles of concentrated sessile dislocations that appear in the regions with large localized strain (figs. S9 and S10). These dislocation bundles, presumably acting as dislocation source, would facilitate dislocation multiplication and slip, giving rise to large local strain.

The deformation microstructure and dislocation density highly depend on crystallographic grain orientation (Table 1). With a single active slip system, grain accommodates the highest density of deformation hcp lamella, resulting from partial dislocation glide on every other plane. Most dislocations are glissile Shockley partials, gliding on parallel planes and scarcely colliding into each other. For the grain of double planar slip, dislocation moving on the intersecting planes results in collision and reaction, leading to formation of Hirth partials and stair-rod dislocations (fig. S5). A notable number of deformation nanotwins appear in the deformed grain, signifying that twin formation and growth are closely related to dislocation interactions. When multiple planes are triggered, the volume density of deformation-induced hcp atoms evolves into the lowest. This reduced propensity for hcp accumulation originates from multiplanar dislocation interactions, which produce various partial dislocations and can cause both hcp layer thickening (fcc to hcp) and thinning (hcp to fcc), depending on the Burgers vector. A dense and intertwined dislocation network, consisting of sessile dislocations and interspersed Shockley partials, emerges from an increased probability of dislocation collision.

**Table 1.** Statistics of deformation structure and dislocation density in grains with one active slip plane, two slip planes, and multiple slip planes. Quantitative characterizations of deformation structure and dislocation at 20% tensile strain.

	**Deformation structures** (volume fraction)				**Dislocation statistics** (density, per m^2^)			
	**Total hcp atoms**	**SF**	**hcp phase**	**TB**	**Total density**	**Shockley**	**Stair-rod**	**Hirth partial**
**Grain 1**	45.2%	16.4%	28.3%	0.5%	6.66 × 10^16^	5.36 × 10^16^	0.45 × 10^16^	3.80 × 10^16^
One active slip plane								
**Grain 2**	31.9%	18%	12.2%	1.74%	2.23 × 10^17^	1.64 × 10^17^	0.23 × 10^17^	0.33 × 10^17^
Two active slip planes								
**Grain 3**	19.2%	15.6%	3.5%	0.1%	1.91 × 10^17^	1.30 × 10^17^	0.14 × 10^17^	0.145 × 10^17^
Multiple active slip planes								

In summary, the study reveals that the maximum strength and critical grain size can be manipulated by tuning the degree of chemical SRO, signifying the nontrivial role of local chemical ordering on the ultimate strength of MPEAs. As the salient feature of MPEAs, the presence of SRO considerably affects deformation microstructure and local plastic strain, originating from its effects on SF energy increase and diffuse antiphase boundary development. As a result, the volume fracture of deformation hcp atoms is decreased to compensate the increased SF energy, and plastic strain is more localized to embrace slip-mediated SRO destruction and glide plane softening. The crystal orientation of constituent grains plays a vital role in deformation mechanisms, microstructure evolution, and dislocation patterning, which collectively affect the mechanical behavior of individual grains and, hence, system-level performance. Moving forward, the emerging MPEAs providing vast compositional space that remains to be explored enable the tunable chemical order at short-range or even medium-range distances, which, together with tailorable grain texture, offer a promising avenue for attaining controllable deformation mechanisms and extraordinary mechanical response beyond the traditional dilute alloys.

## MATERIALS AND METHODS

### MD simulation

We create two independent polycrystal models consisting of 8 and 27 grains to study the grain size effects on strength. To construct different grain-sized systems, the crystallographic grain orientations in the polycrystal are kept the same when scaling the structure and grain to various sizes. A total of 40 samples are created and simulated that contain 0.67 million to 97.32 million atoms and that cover a grain size range of 3 to 40 nm. We use a model embedded-atom method potential (*14*) for CrCoNi alloy, and it enables two control systems, RSS and SRO, for studying the effects of the presence of SRO (SF energy variation and antiphase boundary generation) on deformation mechanisms. With periodic boundary conditions imposed in all three directions, uniaxial tensile deformation is applied to the samples at a constant engineering strain rate of 10^8^ s^−1^ and temperature of 300 K using a Nosé-Hoover thermostat. The mechanical stresses of other directions perpendicular to tension are controlled at zero using a Parrinello-Rahman barostat. The atomistic simulations of deformation are carried out using the open-source code Large-scale Atomic/Molecular Massively Parallel Simulator (LAMMPS) (*29*), and atomic structure visualization and dislocation representation are rendered with Open Visualization Tool (OVITO) (*30*) and dislocation extraction algorithm (*31*).

### MC and MD simulation and SRO

The hybrid MC and MD simulations within the variance-constrained semigrand canonical ensemble (*32*) are performed to construct the systems with chemical SRO. A total of 500,000 MD time steps with a step size of 1 fs are carried out, and for every 50 time steps, the MD simulation is interrupted to execute an MC cycle. In the MC simulations, we carry out *N*/3 number of atom type swap trails, where *N* is the total atoms in the system. In the end of the simulation, each atom, on average, was subjected to 3300 atom type change trials. The acceptance probability is determined by five parameters (*32*), including system energy change ∆*u* after a trail, concertation difference ∆*c* from the targeted equimolar concertation, chemical potential difference between two species ∆μ, variance parameter *k*, and system temperature *T*. We use the parameters ∆μ_Ni − Co_ = 0.021 and ∆μ_Ni − Cr_ = −0.31 eV determined from the semigrand canonical ensemble simulation (*14*), *k* = 1000, and *T* = 650 K, which result in the equimolar concentration in the end of simulation (<0.085 atom % deviation). In the MD intervals, the system stresses are relaxed to zero average value using Parrinello-Rahman barostat. Note that the hybrid MC and MD simulation, incapable of modeling the diffusion kinetics associated with SRO formation as in actual aging experiments, produces an equilibrated state determined by thermodynamics. Nevertheless, it provides a neat low-energy state with SRO, which makes it possible to study local chemical effects on deformation mechanisms by comparing with its counterpart (RSS).

### SRO parameters

To quantify chemical SRO in polycrystals, we modify the nonproportional number (*19*). The order parameter in the first nearest-neighbor shell between any pair of atoms *i* and *j* is defined as, δ*_ij_* = *N_ij_* – *Z*_0_*C_j_*, where *N_ij_* is denotes the actual number of pairs, *Z*_0_ represents the coordination number, and *C_j_* is *j* atom concentration. A positive δ*_ij_* indicates a favored and increased number of pairs, meaning that element *i* tends to bond with element *j*, while a negative value represents an unfavored pairing. For grain atoms, the value of *Z*_0_ is 12 (fcc structure), and *Z*_0_ has a slightly smaller value of 11.42 for grain boundary atoms. The calculated order parameters of grains and grain boundary at the first nearest shell are presented in fig. S2.

### Deformation SF, hcp phase, and TB identification

Dislocation slip and plastic deformation of fcc materials can introduce a variety of hcp-coordinated atomic structures, manifested in the form of iSF, eSF, TB, and hcp phase (lamella). The most frequently used structure characterization methods, such as common neighbor analysis (*33*, *34*) and centrosymmetry parameter analysis (*35*), which can effectively classify the local structure type (for instance, hcp) of each atom, are incompetent at differentiating different hcp structures. To this end, we define a weighted coordination number *Z*, measuring the number of same structure neighbors an atom has within a cutoff distance *r*_c_. When the cutoff distance is covering the two consecutive habit planes [set to be middle of the second (111) and third (111) planes, i.e., $2.5a/\sqrt{3}$; here, *a* is the lattice constant], all the hcp structures, including iSF, eSF, TB, and hcp phase, can be successfully identified from *Z* (*Z*_TB_ = 18, *Z*_eSF_ = 24, *Z*_iSF_ = 30, and *Z*_hcp_ ≥ 37), as shown in fig. S4. The value of *Z* is calculated via coordination analysis of atoms within *r*_c_ and with hcp structure, $f(r_{c}=2.5a/\sqrt{3},\text{hcp})$, and well captures the unique feature associated with each defect, realizing the structure identification.
