# Supplementary material for: Maximum strength and dislocation patterning in multi–principal element alloys
Source: Sci Adv. 2022 Nov 9;8(45):eabq7433. doi: 10.1126/sciadv.abq7433 (PMC9645729; doi:10.1126/sciadv.abq7433)
Supplement: Supplementary file 1 — Figs. S1 to S10 [file sciadv.abq7433_sm.pdf]

Science Advances
AAAS

Supplementary Materials for

Maximum strength and dislocation patterning in multi–principal element alloys

Penghui Cao

Corresponding author: Penghui Cao, caoph@uci.edu

Sci. Adv. **8**, eabq7433 (2022)
DOI: 10.1126/sciadv.abq7433

The PDF file includes:

Figs. S1 to S10
Legends for movies S1 to S3

Other Supplementary Material for this manuscript includes the following:

Movies S1 to S3

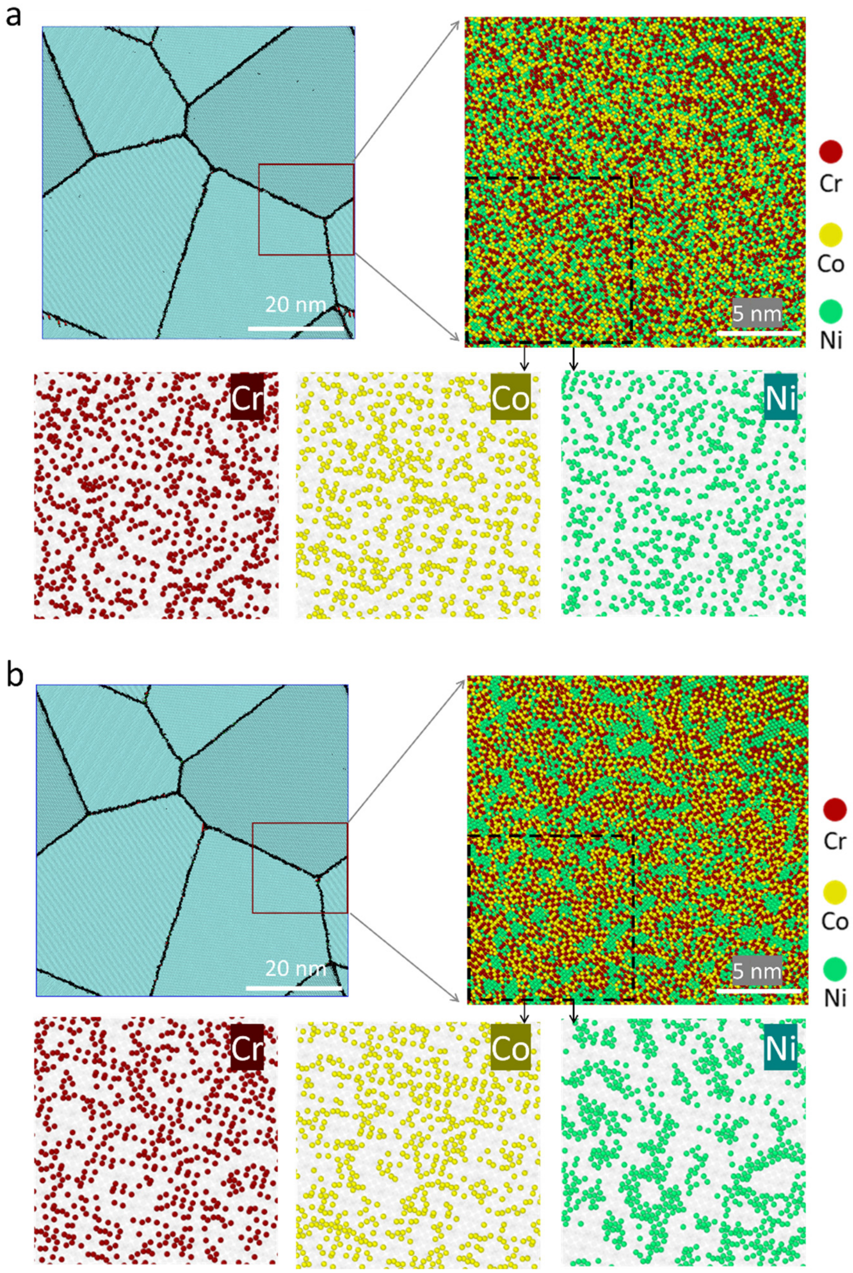

Fig. S1.

Structures and element distributions in RSS (a) and SRO (b) CrCoNi alloys. The bottom panel in each figure, corresponding to the magnification of the local region (black dotted box), shows the concentration distribution of individual elements, Cr, Co, and Ni.

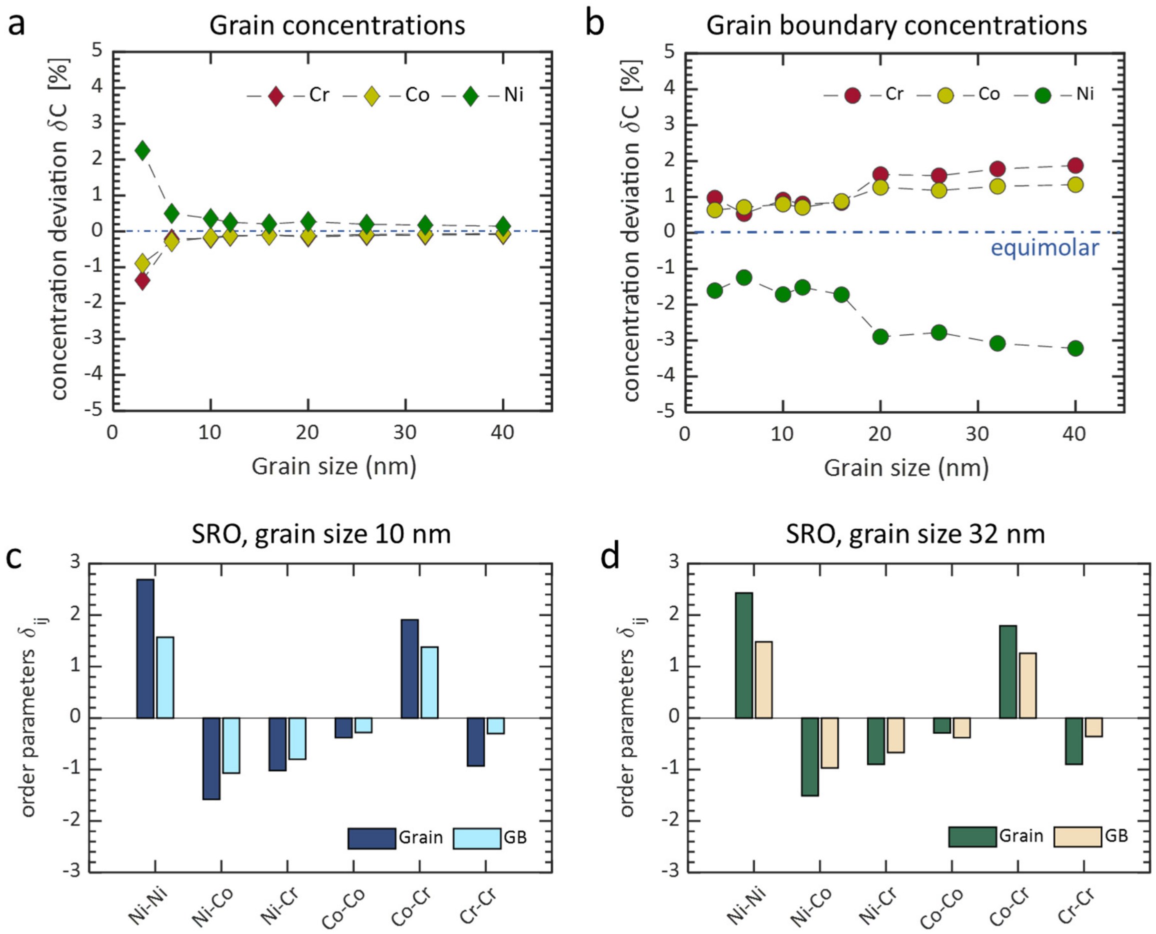

Fig. S2.

Concentration variation and chemical short-range order in annealed polycrystalline alloys. a, Grain concentration for polycrystals with grain size from 40 nm to 3 nm. b, Grain boundary concentration for different grain-sized polycrystals. c,d, Short-range order parameters between a pair of atoms $\boldsymbol{i}$ and $\boldsymbol{j}$, $\boldsymbol{\delta_{ij} = N_{ij} - Z_0 C_j}$, where $\boldsymbol{N_{ij}}$ is denotes the actual number of pairs, $\boldsymbol{Z_0}$ represents the coordination number, and $\boldsymbol{C_j}$ is $\boldsymbol{j}$ atom concentration. The order parameters in grain interiors and grain boundaries are evaluated for annealed polycrystals with the grain size of 10 nm (c) and 32 nm (d).

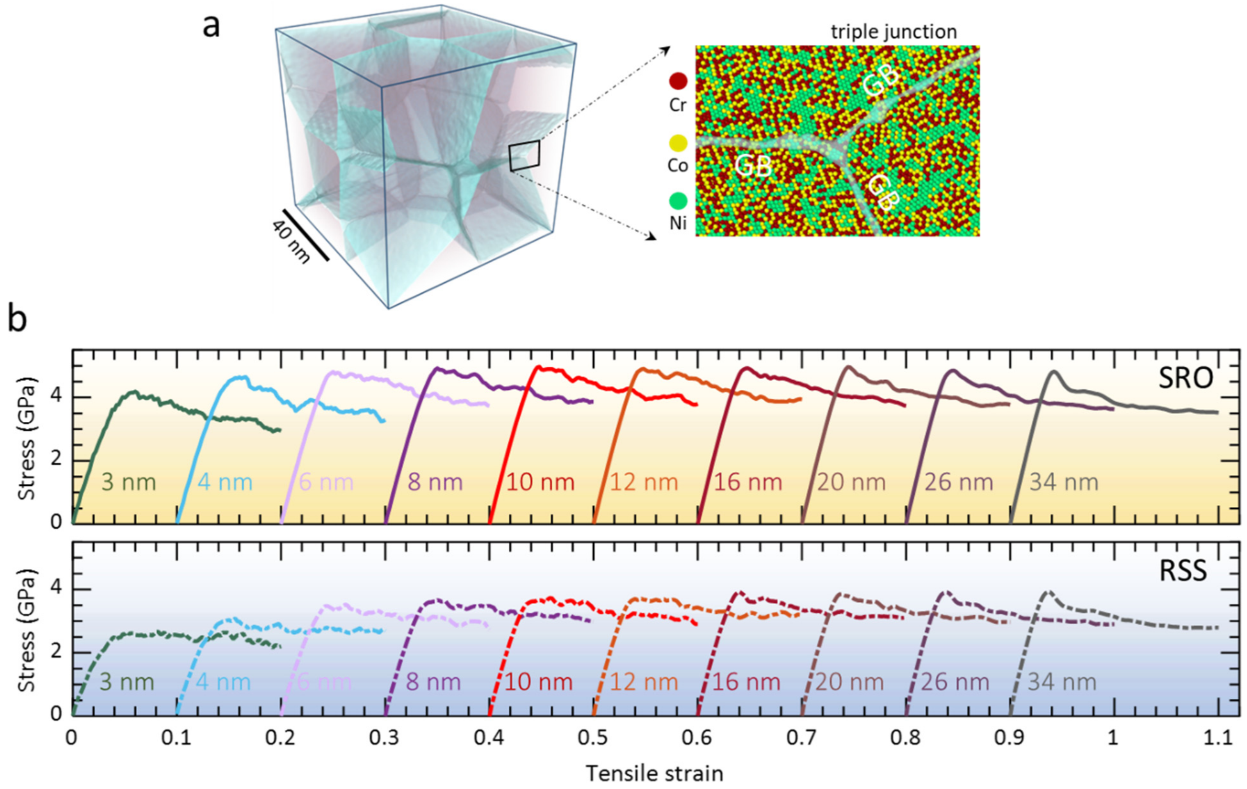

Fig. S3.

Mechanical responses of SRO and RSS systems. a, Thermall-dimensional microstructure of the polycrystalline model and local concentration distributions. b, Stress-strain responses of polycrystals subjected to uniaxial tension. The RSS and SRO denote systems of random solid solution and short-range order, respectively. When comparing these strain-strain curves consisting of an initial elastic regime followed by plastic flow, the flow stress gradually shifts upward with decreasing grain size, but, from about 10 nm, shows an opposite trend with continuous reduction of grain size to 3 nm.

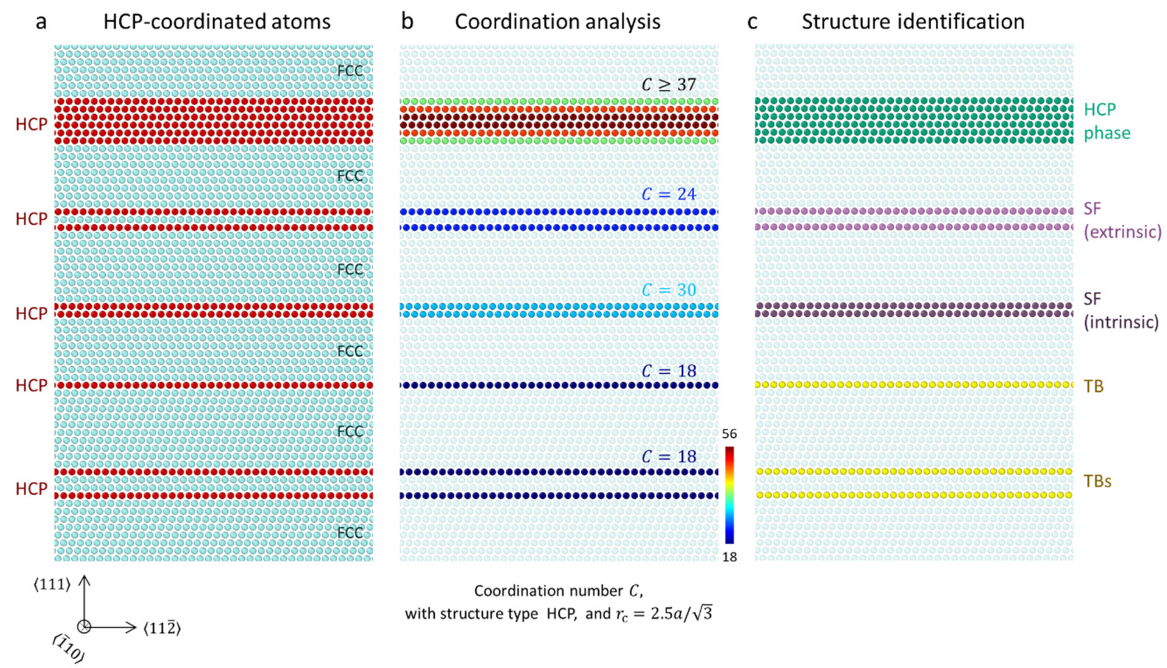

Fig. S4.

Deformation SF, hcp phase, and twin boundary identification. a, A *fcc* structure containing hcp-coordinated atoms. b, Atoms are colored by weighted coordination number Z, that measures the number of same *hcp*-structure neighbors an atom has within a cutoff distance $2.5a/\sqrt{3}$ (*here* a is the lattice constant). c, All the hcp structures, including intrinsic SF (iSF), extrinsic SF (eSF), TB, and *hcp* phase are identified from Z ($Z_{\mathrm{TB}} = 18, Z_{eSF} = 24, Z_{\mathrm{iSF}} = 30, Z_{\mathrm{hcp}} \geq 37$).

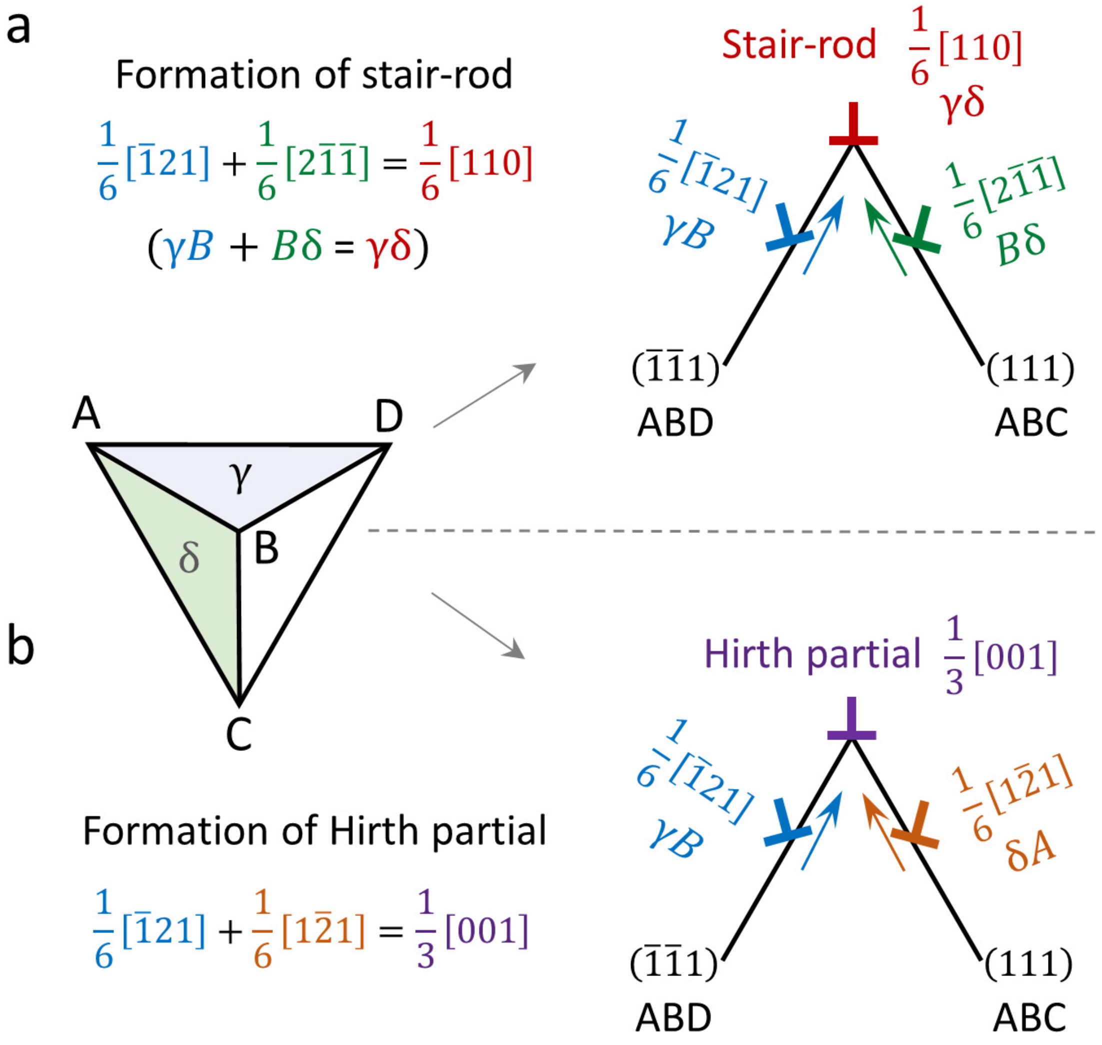

Fig. S5.

Shockley partials interaction and formation of stair-rod and Hirth partial. (a) Two partials $\frac{1}{6}[\bar{1}21]$, $\frac{1}{6}[2\bar{1}\bar{1}]$, collide and produce stair-rod lock, $\frac{1}{6}[110]$. (b) $\frac{1}{6}[\bar{1}21]$ and $\frac{1}{6}[1\bar{2}1]$ partials react and form Hirth lock, $\frac{1}{3}[001]$.

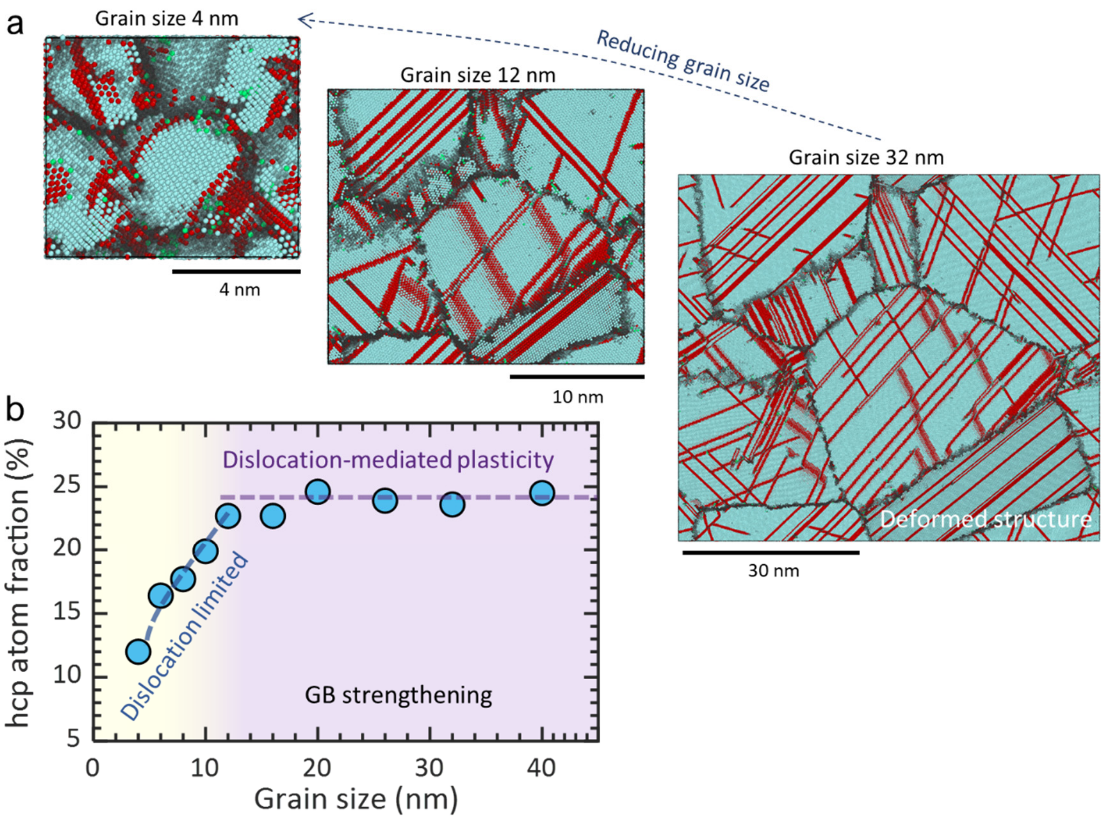

Fig. S6.

Dislocation-mediated plasticity and hcp structure formation. (a) The deformation microstructures (10% applied strain) for systems with grain size 4, 12, and 32 nm, respectively. Atoms are color-coded by structural type. The red color represents hcp-coordinated atoms, and blue indicates fcc structure. (b) The grain size dependence of hcp atom fraction in deformed systems.

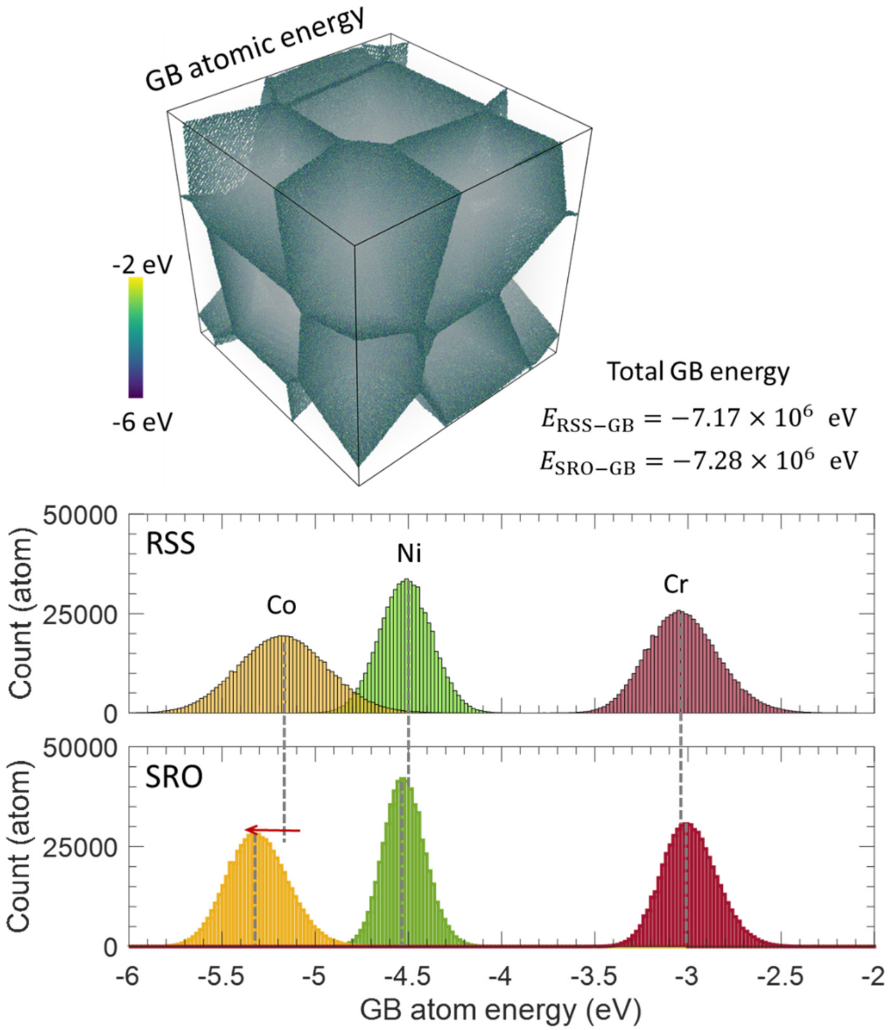

Fig. S7.

A comparison of GB energy in RSS and SRO systems shows the total GB energy is lowered in the presence of chemical order. The top panel shows the atomic energy of grain boundary in polycrystal with a grain size 32 nm. The bottom panel shows the statistical distributions of GB atom energy for RSS and SRO.

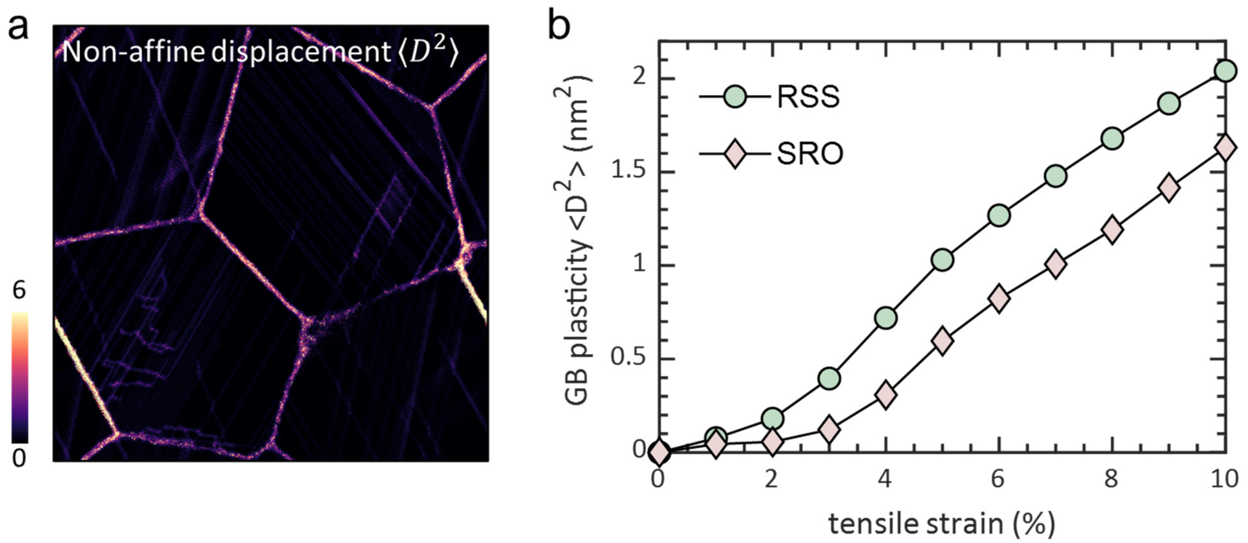

Fig. S8.

Grain boundary (GB) deformation in RSS and SRO. (a) Spatial map of non-affine displacement, D^2, in sample deformed at 10% tensile strain. (b) Non-affine displacement of GB atom as a function of tensile strain for RSS and SRO systems. In the presence of SRO, grain boundary migration and sliding are alleviated.

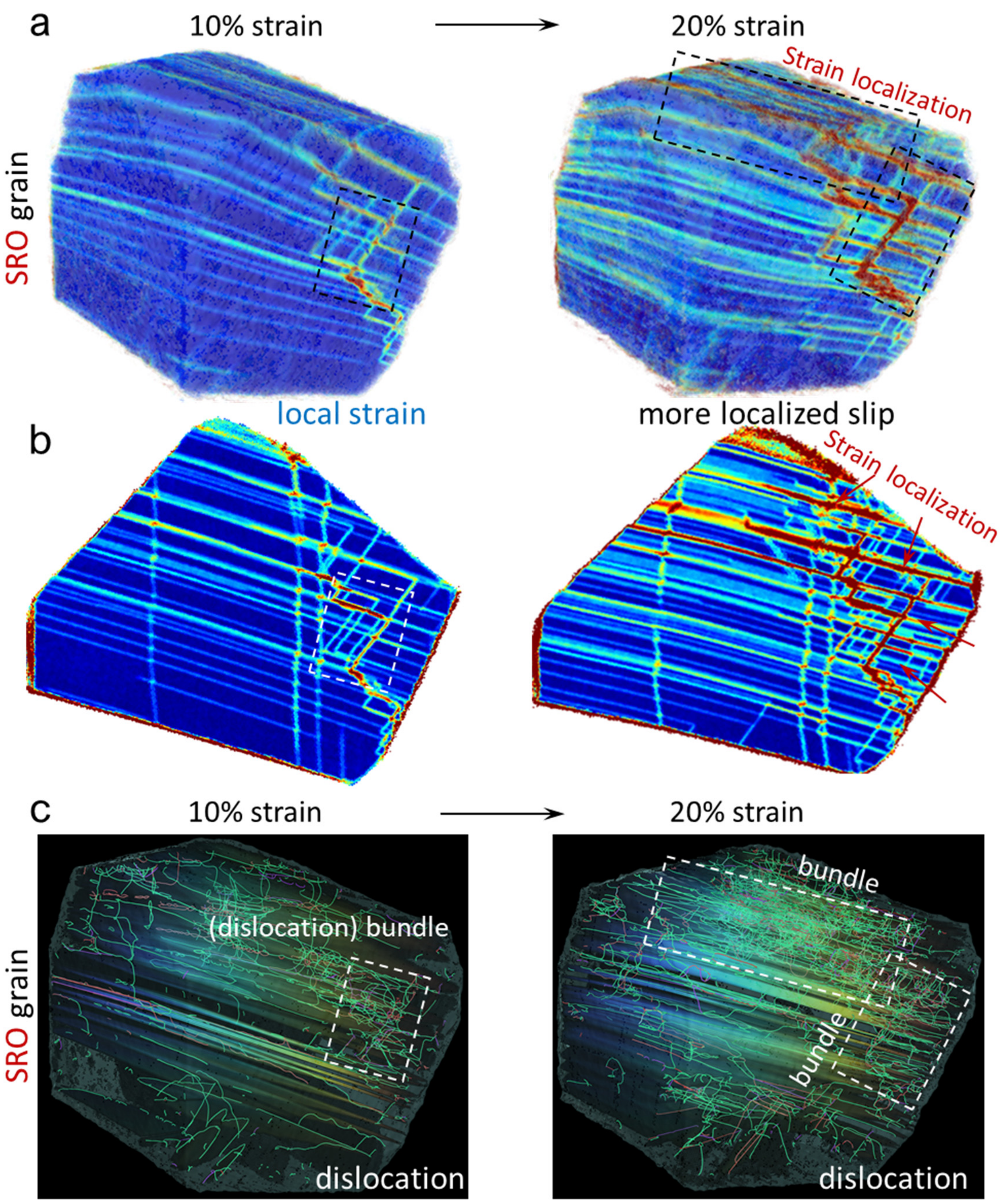

Fig. S9.

Strain localization and dislocation bundle in SRO grain. (a-b) Spatial distributions of local strain at 10% and 20% strain. (c) The corresponding dislocation configurations. Bundle of sessile dislocations appears in the strain localized regions. Green, purple, and red lines indicate Shockley partial, stair-rod, and Hirth partial, respectively.

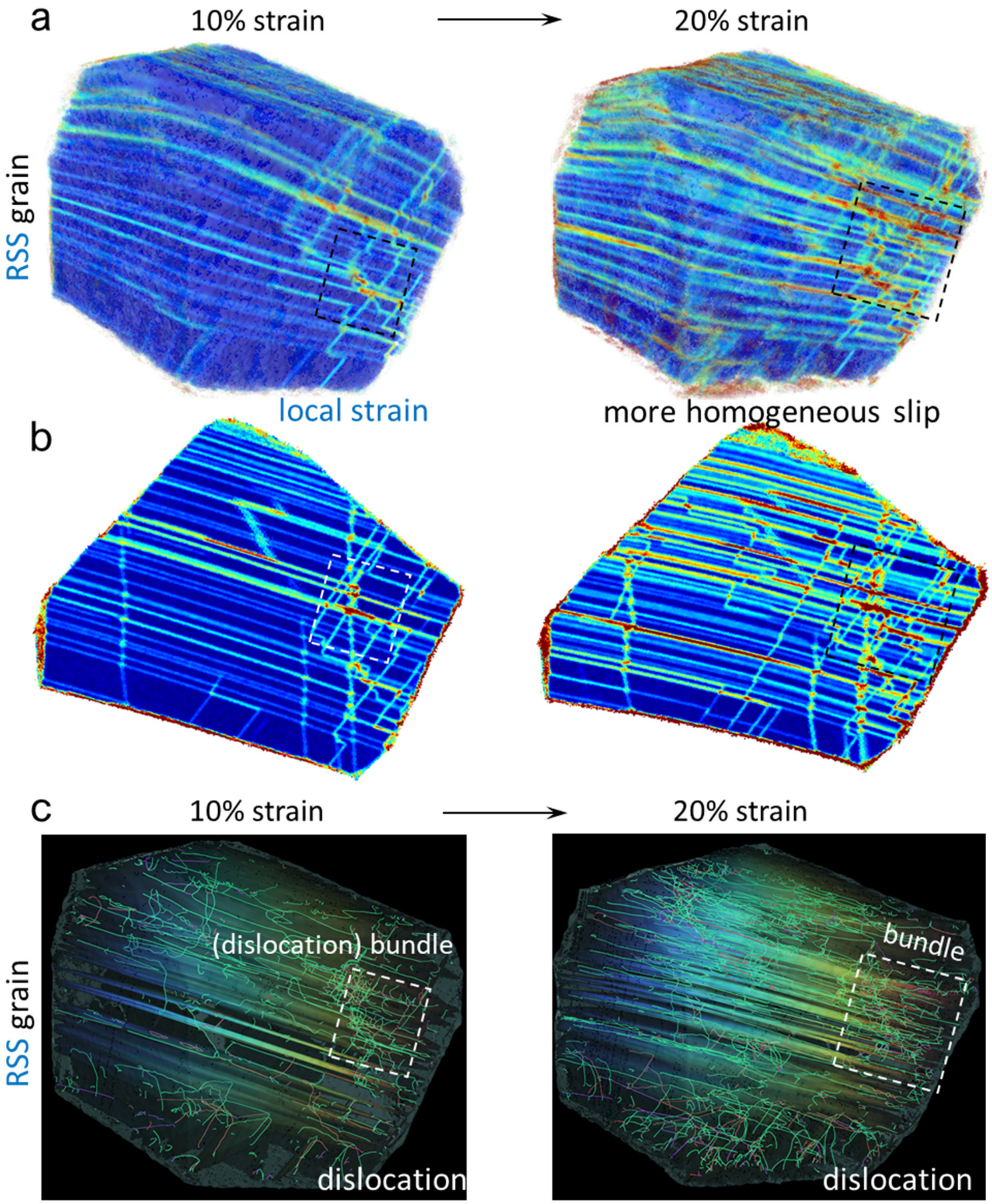

Fig. S10.

Strain localization and dislocation bundle in RSS grain. (a-b) Spatial distributions of local strain at 10% and 20% strain. (c) The corresponding dislocation configurations. Bundle of sessile dislocations appears in the strain localized regions. Green, purple, and red lines indicate Shockley partial, stair-rod, and Hirth partial, respectively.

Movie S1.

Deformation microstructure evolution and dislocation patterning in grain of a single active slip plane. The hcp-coordinated atoms are colored in red. Shockley partial dislocations are green, and stair-rod and Hirth partial dislocations are purple and red, respectively.

Movie S2.

Deformation microstructure evolution and dislocation patterning in the grain of double active slip planes.

Movie S3.

Deformation microstructure evolution and dislocation patterning in the grain of multiple active slip planes.
